# Cigarette Smoking and Alcohol Use among Adolescents and Young Adults with Asthma

**DOI:** 10.1155/2011/503201

**Published:** 2011-12-15

**Authors:** Elizabeth Burgess Dowdell, Michael A. Posner, M. Katherine Hutchinson

**Affiliations:** ^1^College of Nursing, Villanova University, 800 Lancaster Avenue, Driscoll Hall, Villanova, PA 19085, USA; ^2^College of Liberal Arts and Science, Villanova University, 800 Lancaster Avenue, Villanova, PA 19085, USA; ^3^College of Nursing, New York University, 726 Broadway, Room 1047, New York, NY 10003, USA

## Abstract

Asthma is one of the most common, serious chronic diseases in pediatric and young adult populations. Health-risk behaviors, including cigarette smoking and alcohol use, may exacerbate chronic diseases and complicate their management. The aim of this study was to longitudinally analyze rates of cigarette smoking and alcohol use in adolescents and young adults who have asthma and those who do not have asthma. A secondary analysis of data from the National Longitudinal Study of Adolescent Health was undertaken. Individuals with asthma were found to exhibit increasing rates of cigarette smoking and alcohol use as they aged. When an adolescent with a chronic health issue begins health-risk-taking behaviors, behavior change interventions must be planned. Pediatric nurses, practitioners, and clinicians are uniquely positioned to assess for health-risk behaviors in youth with asthma and to intervene with plans of care that are tailored for the needs of this vulnerable population.

## 1. Introduction

Risk-taking behaviors that can have lifelong implications are often begun in adolescence and young adulthood. Two common health-risk behaviors that are frequently initiated are cigarette smoking and the alcohol consumption. The long-term negative health consequences of these behaviors have been well documented, as has their prevalence in the general healthy adolescent population; however, what is beginning to emerge in the literature is that health-risk behaviors are also occurring among youth and adults with chronic health conditions, specifically asthma. This paper discusses the findings from a study that examined cigarette smoking and alcohol use in adolescents and young adults who have asthma and in those who do not have asthma. The sample was drawn through a secondary analysis of data from the National Longitudinal Study of Adolescent Health (Add Health) [1]. Our study addressed the following research questions:

1. How many adolescents and young adults with asthma are engaging in health-risk behaviors (e.g., smoking cigarettes and drinking alcohol)?
2. How do cigarette smoking and alcohol use rates and patterns change as adolescents with asthma age?
3. Are there differences in cigarette smoking and alcohol use behaviors between adolescents and young adults with asthma and those who do not have asthma?

Asthma is a significant and growing public health problem in the United States, with the Centers for Disease Control and Prevention (CDC) estimating that about 7 million children under the age of 18 years are affected [2]. Asthma is considered the most common serious chronic disease in children accounting for approximately 3 million visits to healthcare providers and over 200,000 hospitalizations annually [2, 3]. It is estimated that asthma affects 5% to 15% of the pediatric population with prevalence peaking between the ages of 5–17 years [2, 4]. Further, recent research suggests that the prevalence rates of asthma in children are on the rise; a nearly 72% increase in cases was reported between 1982 and 1994 [3, 5], making pediatric asthma the third leading cause of hospitalizations in those under the age of 18 years.

The course of asthma may vary in the pediatric population with young children, school-age children, and adolescents being managed and managing their asthma and related symptoms differently. Typically younger children rely on a parent or caregiver to monitor their health status, coordinate visits to healthcare providers, and manage medications, including rescue drugs (inhalers, nebulizers, etc.). As children age into adolescence, expectations change such that the monitoring and management of asthma and symptoms are increasingly taken on by the individual adolescent or young adult. At the same time that adolescents may be assuming greater responsibility and control for their own asthma management, they are being faced with the same temptations and motivations that their peers face to engage in “adult-like” health-related risk behaviors. Cigarette smoking is of particular concern as smoking is known to exacerbate asthma and directly contradicts the basic tenets of asthma education and health promotion efforts [6–8].

In the United States, each day approximately 6,000 adolescents, aged 12 to 18 years, try smoking a cigarette for the first time, and 3,000 adolescents become daily smokers [9]. Most of these individuals do so without fully understanding the health risks associated with cigarette smoking both in the short and long term. In addition to being a well-recognized health risk to the general population, cigarette smoking exacerbates asthma and complicates management [6, 8, 9]. Exposure to cigarette smoke is often associated with increased clinical manifestations of asthma symptoms (e.g., wheezing, coughing, and shortness of breath) and an increased need for rescue medications and or medical management [9–11]. Almost all asthma health education materials contain specific references to the hazards of airborne or inhaled irritants and the exacerbation of asthma symptoms by cigarette use, second-hand smoke, wood smoke, or fumes [7, 9]. Yet, contrary to healthcare providers' assumptions and “common sense,” there is growing evidence that individuals with asthma do smoke cigarettes and that their rates of cigarette smoking may be comparable to those of peers who do not have asthma or another chronic illness [6, 12–15].

A large body of research suggests that adolescents who participate in one health-risk behavior are more likely to engage in additional risk behaviors [9, 16, 17]. According to the CDC [9], 75% of high school students report drinking alcoholic beverages at least once. The cooccurrence of alcohol consumption and cigarette smoking documented in the general adolescent population is now being reported among chronically ill youth [18]. The impact of alcohol consumption on lung and airway function is dependent upon concentration, duration, and route of exposure [19]. It has been reported in the literature that prolonged and heavy exposure to alcohol may complicate asthma management in addition to exerting direct negative effects on lung functioning [19]. However this has not been well studied in individuals who have asthma.

Recent studies have shown that adolescents' engagement in health-risk behaviors may be influenced by the social pressures from peers and by their parent role models [6, 20, 21]. Specifically, children who have parents and friends who smoke cigarettes are more likely than others to smoke themselves [20]. Adolescents who smoke cigarettes may also be influenced by parents or guardians who do not supervise the activities of their child. Low parental monitoring has been associated with high school students' reports of smoking cigarettes, using other tobacco products, drinking alcohol, and smoking marijuana [21].

## 2. Materials and Methods

The current study represents a secondary analysis of the public-use data from the National Longitudinal Survey of Adolescent Health (Add Health) [1]. Data were obtained through a contractual arrangement with the proprietors of Add Health [1], and IRB approval was obtained prior to undertaking the secondary analysis. The decision to utilize a secondary analysis for the current study was based on the understanding that, in the nursing literature, the use of “secondary analysis of large databases is increasingly recognized as a valid and efficient method of research” [22]. Using nationally representative databases provides opportunities to address important health issues as these databases allow researchers to study their phenomenon of interest using large samples [22–25].

### 2.1. Procedures

The original study, the National Longitudinal Study of Adolescent Health, is the largest, most comprehensive survey of adolescents ever undertaken in the United States and is based upon a nationally representative sample of adolescents enrolled in grades 7 through 12 in 1994. Add Health contains survey data about adolescents' social contexts (families, friends, peers, schools, neighborhoods, and communities) and how these contexts may influence adolescents' health and risk behaviors. The sampling design for Add Health included complex, multilevel cluster sampling techniques described in detail elsewhere [1, 26–28].

### 2.2. Setting and Participants

Add Health researchers selected a sample of 80 out of the 26,666 high schools in the United States stratified by size, school type, census region, level of urbanization, and percent White. Fifty-two schools agreed to participate, and the remaining 28 were replaced by high schools with similar characteristics. Feeder schools were also identified and included in data collection. Parental consent was obtained prior to data collection [1]. Data for Wave I were collected in 1994-1995 via in-school student questionnaires, school administrator questionnaires, and in-home interviews with students and one of the student's parents (usually the mother). The in-home interview data with students and parents were collected in 1995. A second wave (Wave II) of data was subsequently collected in 1996 (not used in our analyses); a third wave of data (Wave III) was collected in 2001-2002. Respondents, aged 18 to 26 years at the time of Wave III, were reinterviewed to investigate their transitions to young adulthood.

The sample for our study's secondary analysis consisted of 4,882 adolescents and young adults who participated in the Wave III survey. Eight cases from Wave III were subsequently excluded due to missing data for whether or not they had asthma, resulting in a final analytic sample of 4,874 individuals.

### 2.3. Data Collection and Analysis

All analyses were conducted using SAS version 9.1 (SAS Institute, Cary, NC). For this analysis, cross-sectional sampling weights for Wave III were used on all data. Chi-squared tests were used for comparisons of categorical data, and independent two-sample *t*-tests were used for continuous/numeric variables. Adjustments for sampling designs were made using the Rao-Scott method [29]. Analyses were adjusted for clustering and weighting from the publicly available dataset, but did not address the stratification, as that variable was not available in the public-use dataset. However, it has been reported that exclusion of the strata variable in the public dataset “only minimally affects the standard errors” [30].

### 2.4. Measures

The current study employed measures from the Wave I and Wave III questionnaires. Demographic measures from adolescents included Wave I reports of age, gender, race, ethnicity, and household composition. Background information from parents (usually the mother) at Wave I included age, marital status, asthma, smoking, and alcohol use.

Key measures of interest in this study included questions from Waves I and III asking about asthma status, cigarette smoking, and alcohol intake. Asthma status was assessed via self-report by asking, “Have you ever been diagnosed with asthma?” Participants responded “yes” or “no.” Cigarette smoking and alcohol consumption were assessed with two sets of questions. The first asked participants whether they had “ever” tried the behavior (smoking cigarettes or drinking alcoholic beverages). Second, participants were asked if they had “regularly” smoked (defined as at least one cigarette per day for 30 days). The response choices were “yes” or “no.” Participants were also asked to rate how much alcohol they drank. The question was worded, “Think of all of the times you have had a drink in the past twelve months. How many drinks did you usually have each time?”

## 3. Results

A total of 818 (16.2%) Add Health participants were self-identified as having asthma and were included in the asthma group; 4,056 were self-identified as not having asthma and were included in the nonasthma group. As is shown in Table 1, there were no significant differences in the two groups in terms of age, sex, or race. The asthma group included slightly more females, although these differences were not statistically significant. However, those in the asthma group were more likely to speak only English in the home and were less likely to have been born outside the USA compared to those in the nonasthma group (*P* < .01 and *P* < .05, resp.). Those with asthma reported having more health problems related to allergies when compared to their nonasthma peers (70% versus 37%; *P* < .001) and were more likely to have a family history of asthma (*P* < .001); 19% of the asthma group versus 7% of nonasthma group had a biological mother with asthma and 15% of the asthma group versus 5% of the nonasthma group reported having a biological father with asthma (*P* < .0001 for both).

### 3.1. Smoking Cigarettes

Findings from Wave III indicated that those with asthma had higher rates of having tried cigarette smoking (79%) compared to those without asthma (75%; *P* < .05). In addition, those in the asthma group were more likely to become later smokers than those in the nonasthma group. Comparing smoking patterns at Wave I and Wave III for both groups found that there was an increase of 18% in smoking within the asthma group (they reported not smoking in Wave I but responded “yes” to regularly smoking eight years later in Wave III). As is shown in Table 2, there was additional evidence (*P* < 0.06) that suggested those with asthma were more likely to smoke regularly than those without asthma (72% versus 67%). Those in the asthma group (33%) were also more likely to report experiencing feelings of moderate or intense physical relaxation when they began smoking compared to 29% of the nonasthma group (*P* < 0.05).

### 3.2. Drinking Alcohol

Participants in the asthma group reported drinking behaviors that were comparable to those without asthma at both Wave I and Wave III. There were no significant differences between the two groups' reports. However, differences were found between the groups in changes in the amount of alcohol consumption that occurred between Waves I and III. As is shown in Table 3, the asthma group drank 0.22 drinks less than their nonasthma peers at Wave I (early- to mid-adolescence); six-seven years later (Wave III; young adulthood), the asthma group reported drinking 0.30 drinks more than those in the nonasthma group. This represented an increase of almost one full drink per episode.

In addition, participants in the asthma group were more likely to report alcohol-related risk behaviors at Wave III, compared to those in the nonasthma group. Specifically, participants with asthma were more likely to report having at least one best friend who was a binge drinker (5+ drinks on a single occasion) (58% versus 55%; *P* < 0.05). Asthma group participants also reported being slightly more likely to report having a sexual situation occur after drinking alcohol than the group without asthma (24% versus 21%); this finding was worrisome although not statistically significant.

## 4. Discussion and Clinical Implications

Three major findings emerged from this study: (1) adolescents and young adults with asthma are smoking cigarettes and drinking alcohol; two behaviors that are contraindicated for individuals with asthma; (2) the asthma group participated in these health-risk behaviors at rates that were similar to their nonasthma peers during adolescence and at even higher rates during young adulthood; (3) those in the asthma group reported elevated rates of lifestyle risk behaviors associated with alcohol use. The study's findings have implications for nurses, nurse practitioners, health educators, and other professionals who work with adolescents and young adults who have asthma.

When a diagnosis of asthma is made, healthcare professionals begin anticipatory guidance related to health promotion and management of asthma symptoms. Asthma education focuses on identifying triggers, recommendations for a healthy lifestyle, limiting exposure to allergens, use of medications (rescue and maintenance), and adherence to a holistic plan of care. Goals of asthma therapy include minimal or no chronic symptoms (daytime or nighttime); minimal or no exacerbations; no limitations on physical activities (no missed school or work days); minimal use of short-acting inhaled Beta2-agonists; minimal or no adverse events from medication [2, 9, 31]. Successful treatment of asthma may be impeded when other risk behaviors are present particularly in the face of severe asthma [6]. Smoking cigarettes is recognized as a deterrent to healthy pulmonary function and would be contraindicated in any plan of care for asthma.

Adolescent cigarette smoking can be viewed within a broad profile of risk factors that incorporate the desire to take health risks, peer influences, parental role modeling, environment, perception of current health, and any chronic conditions [6, 20, 32, 33]. To better assess individuals with asthma and gauge their participation in health-risk behaviors, ongoing risk behavior assessments should be conducted at each healthcare visit. Pediatric nurses, practitioners, clinicians, and other healthcare professionals are in an ideal position to assess the type of and level of participation in an adolescent or a young adult with asthma. Using open-ended questioning is ideal for exploring what experiences adolescents have had with cigarettes or alcohol, how long they have been smoking or drinking, how the behavior began, where they are encountering cigarettes or alcohol, how they perceive their friends' participation with these substances, and what behaviors they see in their household with their parents.

In addition to assessment, a family-centered approach to asthma management is important. Whether or not the parents or other household members smoke needs to be assessed [6, 7]. The potential influence of parental monitoring/supervision, role modeling of risk behaviors, and access to cigarettes and alcohol in the home should be discussed with parents. Education on risk reduction strategies and referrals for smoking cessation assistance should be offered when indicated.

Reasons for smoking and how cigarettes make adolescents feel should also be assessed. For example, in the current study those with asthma reported feeling a physical relaxation from smoking and were less likely to report having tried to quit smoking cigarettes. Asking questions during an exam or taking a history specific to cigarette smoking, age of first cigarette, how many cigarettes they smoke a day, how smoking makes he or she feel, and if they have tried to quit smoking may provide insight that can direct the development of an individualized plan of care to decrease or cease cigarette smoking.

Drinking alcohol was also found to be a health-risk behavior for the asthma group in this study. As young adults the asthma group reported drinking slightly more alcohol than those without asthma. In addition, they reported somewhat higher rates of risky lifestyle behaviors associated with alcohol use. Having a friend who binge drinks, participating in a sexual situation after drinking alcohol, and being drunk at school or work (8% of the asthma group versus 6% of the without asthma group) are behaviors that together or alone raise alarms. For example, adolescents without a chronic illness who have alcohol-related disorders or heavy consumption of alcohol have been found to be more likely to smoke cigarettes, less likely to exercise, and less likely eat a balanced diet and take vitamins compared to those without alcohol use disorders [33]. Further study is needed to better understand the participation in risky lifestyle choices associated with drinking alcohol and how these are affecting individuals with asthma, their level of health and disease knowledge in addition to their commitment to a wellness lifestyle. Research into how much alcohol is being consumed by adolescents and young adults with asthma and how their alcohol intake may relate to their lung functioning, occurrence of respiratory infections, and use of asthma medications is also essential.

Alcohol use and cigarette smoking are complex behaviors whose prevalence appears to increase with age in the general population of adolescents and young adults [2]. Our study findings support this literature with similar findings in an at-risk population, adolescents and young adults who have asthma. It is not uncommon for youth with or without a chronic illness to want to belong and behavior as others. There is a growing body of research that has found youth with a chronic condition may have additional risks for engaging in health-risk behaviors (e.g., smoking cigarettes, smoking marijuana, performing violent, or antisocial acts) when compared with healthy peers [6, 34, 35]. Our study furthers the existing literature by comparing cigarette smoking and alcohol consumption between those with and without asthma and by examining these behaviors longitudinally across adolescence and the transition to young adulthood. That our study found an increase in smoking cigarettes and drinking alcohol in an asthma population suggests that continued education and health promotion by nurses and other healthcare professionals is imperative during this transitional time period.

The diagnosis of asthma in our study's population was made before the 7th grade, a finding that implies that the child's parent or guardian was aware of the condition. In asthma education and management parents or guardians are involved in every aspect of health and treatment when their children are young. As children age into adolescence, asthma management may gradually shift from parent to adolescent and increase the youth's accountability and responsibility for his or her disease and management. Nonetheless, strong parental support of positive behaviors that promote health has been found to be associated with lower rates of risk behaviors in adolescents with health issues [17].

In this study behaviors of cigarette smoking and drinking alcohol were found during adolescence and then again six to seven years later during the young adult time period. Pediatric nurses have an influential role in how adolescents manage their health, asthma management, and risk taking behaviors as they age. Providing positive anticipatory guidance and including both adolescents and parents in the plan of care may have a better chance of succeeding in promoting healthy lifestyle choices that will be with them across their lifespan.

Nurses and other healthcare providers must consider directing their health promotion and educational efforts towards two areas with an asthma population: prevention and smoking cessation. Nurses can find time during an individual's primary health visit, specialty clinic visit, school nurse visit, parish visit, community or home visit to convey health promotion information that is focused on the prevention and abstinence from smoking. There are currently numerous cessation programs designed to educate individuals about the hazards of cigarette smoking and drinking alcohol for healthy adolescents and young adults. Nurses, practitioners, educators, and other professionals are in ideal positions to create new programs or modify existing ones to incorporate interventions and materials that address the special needs of a young, chronically ill population.

## 5. Research Implications

We do not fully appreciate the relationships between the developmental needs of the adolescents, the physiology of their health condition, and social influences of family and peers. Health-related behavior is complex, involving a myriad of factors that contribute to the occurrence of health promotion and health-risk behaviors that can begin in adolescence and continue into adulthood. Findings from this study are consistent with the broader literature on health behavior that highlights that knowledge alone and a diagnosis of asthma may not account for behavior or act as factors in preventing smoking [6, 7, 31, 35, 36]. Our study supports findings from Tercyak using Add Health Wave II data [35]. Tercyak reported that adolescents with asthma were just as likely to experiment with smoking in their lifetime and were significantly more likely to be current smokers than their peers without asthma. Further, rates of current smoking were higher in the asthma group (48%) compared to those without asthma (42%). Given the current study findings that smoking rates continued to increase as youth with asthma aged, equal to or exceeding rates in the nonasthma group, further research should focus on understanding the factors that contribute to the adoption of heath-risk behaviors of this chronically ill group.

## 6. Limitations

The study findings should be viewed in light of the study limitations. Two of the most significant shortcomings in the current study relate to the use of abbreviated measures in the Add Health dataset and the omission of several clinically relevant variables. Specifically, this study relied upon adolescents' self-reports of having asthma. No confirmatory medical diagnoses were obtained, and no data regarding the severity of the asthma (e.g., mild, moderate, or severe) were collected. In addition, there were no data collected regarding the type of asthma (e.g., reactive airway, physical exertion, or allergy induced). These two shortcomings are commonly encountered limitations in secondary analyses with large, national datasets [23–25, 37]. While these types of datasets provide large, representative samples, and breadth of data, they tend to lack detailed measures in any single area [23–25, 37]. Nonetheless, the Add Health study data were well suited to this initial examination of cigarette smoking and alcohol use among adolescents and young adults with asthma.

## Figures and Tables

**Table 1:** Demographic characteristics of the sample at Wave III.

	Total sample	Asthma group	Without asthma group
*N*	4,874	818	4,056
Age (years)	21.8 (1.8)	21.7 (1.8)	21.8 (1.9)
Gender			
Male	50.7%	48.8%	51.1%
Female	49.3%	51.2%	48.9%
Race/ethnicity			
White	77%	77%	77%
Black	16%	16%	16%
Black and White	0.5%	0.7%	0.5%
Other races (non-Black, non-White)	7%	6%	7%
Speak language other than English at home	7%	4%	7%**
Born outside of U.S.	5%	4%	6%*
Have health problems related to allergies		57%	3%***
Biological mother with asthma		19%	7%***
Biological father with asthma		15%	5%***

* *P* < .05; **P < .01; ***P < 0.001.

**Table 2:** Smoking cigarettes: asthma group and without asthma group at Wave III.

	Asthma group	Without asthma group
*N*	818	4,056
Ever tried cigarette smoking	79%	75%*
Ever experienced moderate/intense relaxation when smoking	33%	29%*
Ever smoked cigarettes regularly	72%	67%^#^

^#^ *P* < .06; *P < .05.

**Table 3:** Drinking alcohol: asthma group and without asthma group at Wave III.

	Asthma group	Without asthma group
*N*	818	4,056
Number of drinks per time	4.75	4.53
Have at least one of three best friends who binge drink	58%	55%*
Number of times experiencing a sexual situation later drinking at least once	24%	21%

*P < .05.
